# Feasibility of contextualizing the Informed Health Choices learning resources in Italy: A pilot study in a primary school in Florence

**DOI:** 10.12688/f1000research.123728.1

**Published:** 2022-10-12

**Authors:** Camilla Alderighi, Raffaele Rasoini, Giulio Formoso, Maria Grazia Celani, Sarah E. Rosenbaum

**Affiliations:** 1 Associazione Alessandro Liberati Cochrane Affiliate Centre, Lauria, Italy; 2 Azienda USL IRCCS di Reggio Emilia, Reggio Emilia, Italy; 3 Cochrane Neurological Science Field, Perugia, Italy; 4 Direzione Regionale Salute, Regione Umbria, Perugia, Italy; 5 Centre for Epidemic Interventions Research, Norwegian Institute of Public Health, Oslo, Norway

**Keywords:** Critical thinking, evidence-based medicine, informed health choices, critical health literacy, health literacy, public health

## Abstract

Background

The Informed Health Choices (IHC) project team developed learning resources for primary school children to teach critical thinking about treatments claims and health choices and evaluated their effect in a randomized controlled trial of 120 schools in Uganda. Children taught with these resources showed a better ability to think critically about treatments claims and health choices than children not taught with these resources. Teams in multiple countries are contextualising the IHC resources for use in other languages and settings; in this pilot we describe contextualization for use in Italian primary school.

Methods

After translating the IHC resources to Italian and holding an introductory workshop with participating schoolteachers, we piloted the resources with two classes of a primary school in Florence over nine lessons. Our aims were: 1) to assess the feasibility of introducing the IHC curriculum in Italian primary school; 2) to evaluate students’ ability to assess health claims and make informed health choices; to explore 3) students’ and 4) teachers’ experiences with the IHC learning resources; 5) to identify barriers and facilitators to implementation of IHC learning resources in Italian primary school. To assess these objectives, we used qualitative and quantitative methods.

Results

Both qualitative and quantitative analyses consistently showed that the IHC learning resources had a positive impact on the objectives examined. The resources integrated well into the Italian primary school curriculum. Both students and teachers considered these resources comprehensible, appealing in design and content, and stimulating for the development of a critical attitude. The only barrier teachers and students expressed was using the resources in a remote learning context.

Conclusions

Findings from our contextualisation of IHC learning resources in Italian primary school indicate that these resources are well-suited for Italian teachers and students in a primary school context and compatible with the Italian primary school curriculum.

## Introduction

People’s desire and demand to be involved in health decisions have increased progressively in the last few decades. ^1^ In a systematic review of 115 studies on patients’ decision role preferences for treatment and screening, most patients preferred sharing decisions with physicians in 63% of the studies. This represents a shift from an earlier model, where doctors make most of the decisions about patients’ health, to a shared decision model where patients participate more actively.

A first step for a person who wants to participate in their own health decisions is to be able to access reliable information about the treatment effects of options that are relevant to them. There is no lack of information about treatments - claims about “what works” come from a wide variety of sources, such as healthcare professionals, acquaintances, relatives, blogs, TV, social media, scientific magazines, and the lay press. Despite this abundance of information, most claims about health interventions that people hear or read about every day are far from reliable. ^2^^,^^3^ Easily accessible claims published on the internet, on TV or in the lay press are often not based on sound scientific evidence; even if they are, they can be reported in incomplete or misleading ways. ^4^

Physicians and scientists are not exempt from making unreliable health claims. For example, physicians often underestimate the risks and overestimate the benefits of diagnostic and therapeutic interventions. ^5^ This biased perspective can lead to unbalanced conversations with patients and tend to favour interventions’ advantages over disadvantages. ^6^ It is challenging for people to make good health choices when they are subjected to an overabundance of unreliable or unbalanced information, and they need skills to be able to navigate this landscape.

Health literacy is the degree to which individuals can find, understand, and use information and services to make informed health decisions for themselves and others. ^7^ But health literacy skills are lacking, both in Europe and more specifically in Italy. In a European survey, 47% of the adult population had problematic or inadequate self-perceived health literacy; for the Italian adult population this number increased to 54%. ^8^

In response to the importance of this problem and the widespread lack of skills to address it, a group of physicians, teachers, public health experts, epidemiologists, designers, and journalists developed the Informed Health Choices (IHC) project in 2012. Their objective was to improve people’s ability to think critically about health choices, starting with primary school children. ^9^

Whereas learning new concepts in adulthood can be hindered by prejudices, misconceptions, and entrenched personal narratives, children have a more open and flexible approach towards learning. Moreover, children have been found to be able to learn the bases of critical thinking since primary school. ^10^ Teaching critical thinking to primary school children can prepare them for making informed and unbiased choices as adults.

Using a human-centred design approach, ^11^ the IHC group developed learning resources that aimed at teaching primary school children how to evaluate health claims and make informed health choices. Their first step was to develop a group of Key Concepts that established what students needed to learn to develop these skills. These concepts also provide a map to orient people towards critical thinking both in medicine and other knowledge areas. ^12^ The full set of IHC Key Concepts (reviewed and updated yearly since 2012 and published at thatsaclaim.org) are classified into three thematic areas: claims, evidence, and choices: how to assess the reliability of health claims about treatment effects; what characterises reliable evidence from health research; and how to make informed health choices using the information that is available to you.

**Figure 1.**
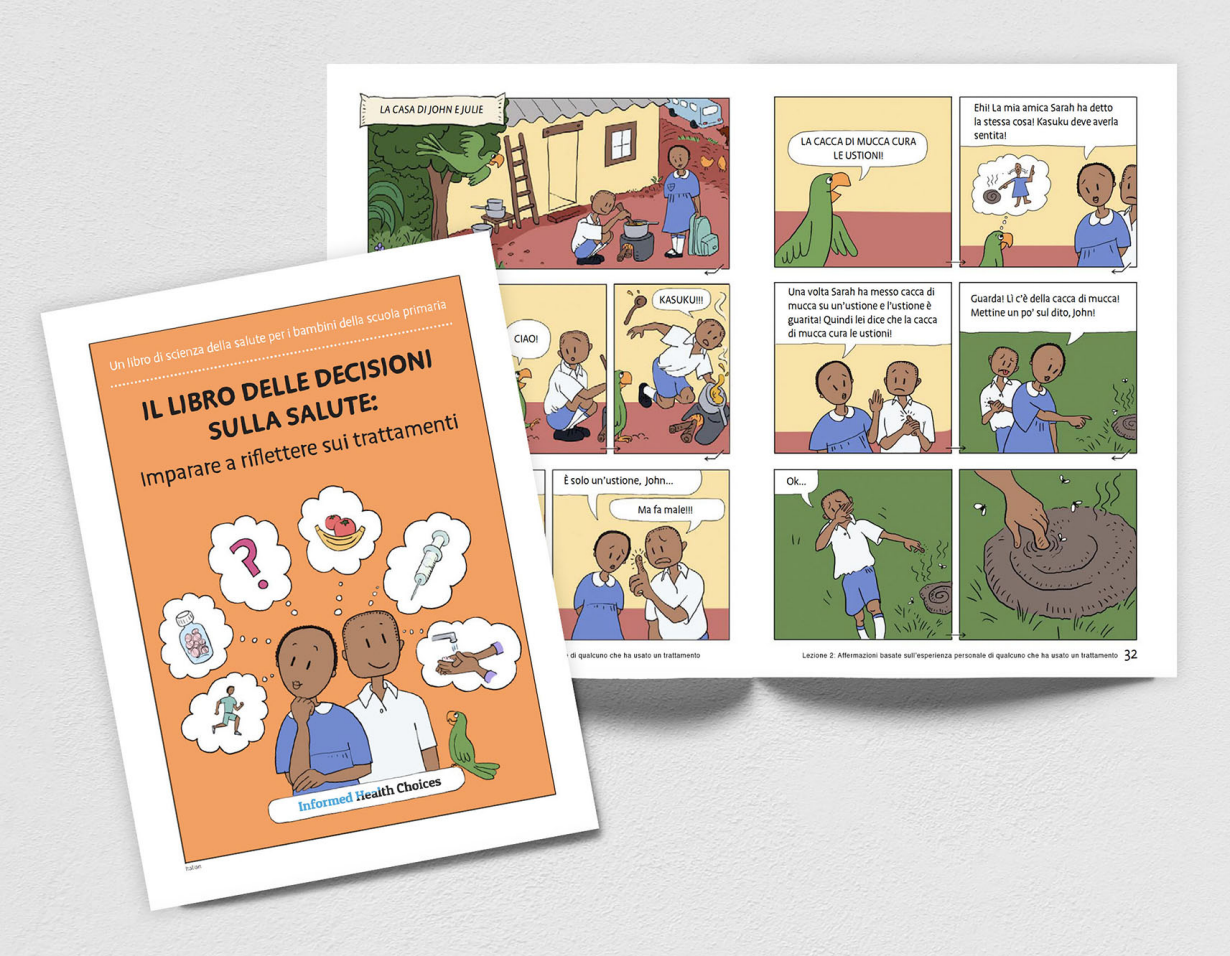
Italian translation of “The Health Choices Book: Learning to think carefully about treatments” for primary school children.

**Figure 2.**
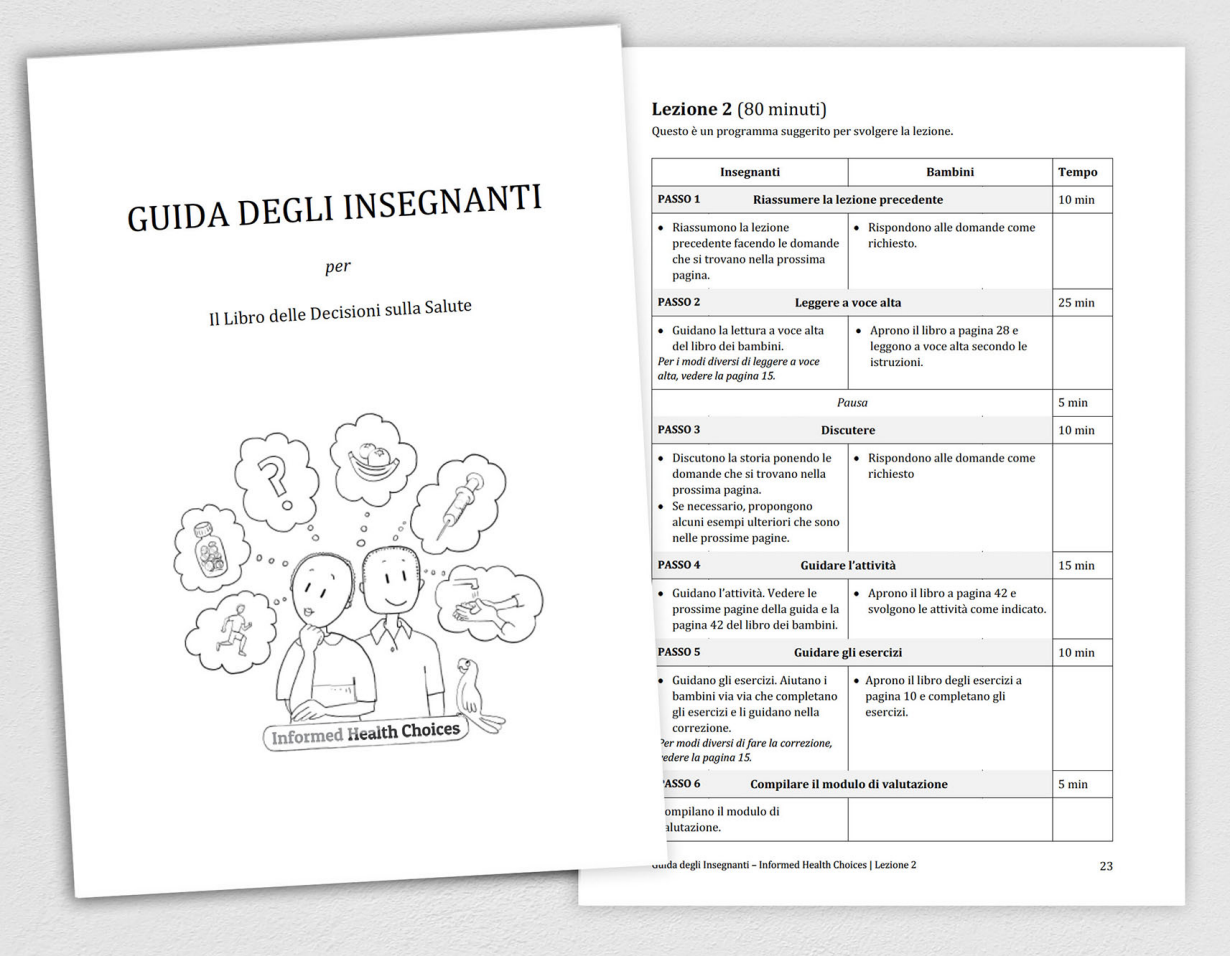
Italian translation of “Teachers’ Guide for The Health Choices Book”, for primary school teachers.

**Figure 3.**
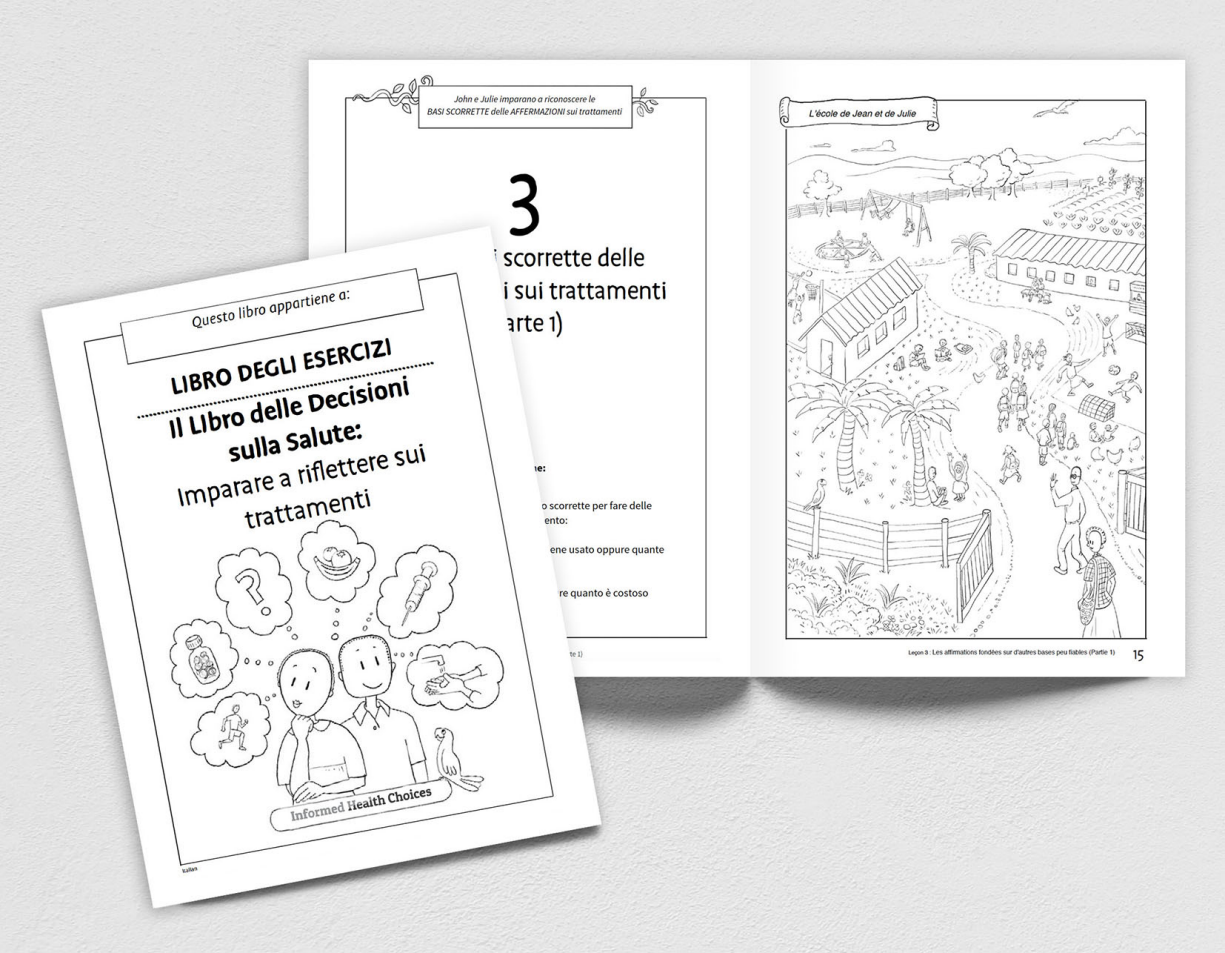
Italian translation of “Exercise Book for The Health Choices Book”, for primary school children.

Among the 49 Key Concepts, 12 were selected by the IHC group to be the basis for the resources for primary school children, age 10-12 years ( Table 1). The main resources for teaching are the *Health Choices Book*, *Teachers’ Guide*, *Exercise Book* ^13^^,^^14^ ( Figures 1- 3).

**Table 1.** The 12 Key Concepts that are taught in the IHC primary school resources.

*Main concept group*	*Key Concept*
Recognising **claims** about the effects of treatments that have an unreliable basis	*Treatments may be harmful*
	*Personal experiences or anecdotes (stories) are an unreliable basis for assessing the effects of most treatments*
	*Widely used treatments or treatments that have been used for a long time are not necessarily beneficial or safe*
	*New, brand-named, or more expensive treatments may not be better than available alternatives*
	*Opinions of experts or authorities do not alone provide a reliable basis for deciding on the benefits and harms of treatments*
	*Conflicting interests may result in misleading claims about the effects of treatments*
Understanding whether **comparisons** of treatments are fair and reliable	*Identifying effects of treatments depends on making comparisons*
	*Apart from the treatments being compared, the comparison groups need to be similar at the beginning of a comparison (i.e. ‘like needs to be compared with like’)*
	*If possible, people should not know which of the treatments being compared they are receiving*
	*Small studies in which few outcome events occur are usually not informative and the results may be misleading*
	*The results of single comparisons of treatments can be misleading*
Making informed **choices** about treatments	*Decisions about treatments should not be based on considering only their benefits*

After being taught with the Key Concepts, students are requested to fill in a final test—questions from the “Claim Evaluation Tool”. This is a flexible battery of multiple-choice questions designed to assess understanding and use of the Key Concepts, iteratively developed and validated for use by children and adults across high- and low-income settings. ^15^^,^^16^

The IHC project team evaluated the impact of the school resources in a cluster randomized trial involving 120 primary schools in Uganda. ^17^ The trial—published in 2017—found that 10- to 12-year-old children who were taught lessons from the *Health Choices Book* that covered 12 Key Concepts, developed a better critical and decisional attitude about health claims and treatments than those who did not use these learning resources. Moreover, a follow-up of this trial found that children retain the ability to assess health claims for at least one year after the end of the lesson cycle, ^18^ and a process evaluation showed that the resources were highly valued by both teachers and students. ^19^

The core IHC group also developed contextualization guidance, to support subsequent translation and adaptation of learning resources to contexts that differ from where they were developed and tested in East Africa. ^20^^–^^22^ Several research teams around the world have since engaged in contextualisation activities, with the aim of assessing the feasibility of resource application in different contexts.

In 2019, two authors of this article (CA and RR) started the Italian translation of the IHC learning resources and initiated a pilot study at a public primary school (Matteotti Primary School, Poliziano Institute) in Florence, Italy, in 2020. This study was designed to explore this contextualization of the IHC learning resources in Italy.

### Objectives

Our primary study objective was to assess the feasibility of introducing the IHC curriculum in Italian primary schools. Our secondary objectives were to evaluate the ability of students in the pilot to assess health claims and make informed health choices, to explore students’ and teachers’ experiences of the learning resources, and to identify barriers and facilitators to implementation of the IHC curriculum in Italian primary schools.

## Methods

We carried out two main contextualisation activities: translation of the IHC learning resources to Italian and piloting of these translated resources in an Italian primary school.

### Translating resources

The objective of the translation activities was to ensure that the language used in the IHC learning resources was suitable for use in Italian primary schools. We translated the learning resources from English into Italian from February to November 2019, following guidance from the IHC group, ^20^^,^^22^ guidance for cultural adaptation ^23^ and for translating and adapting tests. ^24^ CA and RR independently translated the *Health Choices Book* and *Exercise Book.* We then reviewed and collated these into a single draft, making further adjustments after comparing it to the French and Spanish translations, based on linguistic analogies with Italian language. We collected feedback in two rounds, described under ‘Data collection’.

### Piloting translated resources

For the pilot, we had five objectives: 1) to assess the feasibility of introducing the IHC curriculum in Italian primary school, 2) to evaluate pilot students’ ability to assess health claims and make informed health choices, to explore 3) students’ and 4) teachers’ experiences of the IHC learning resources 5) to identify barriers and facilitators to implementation of the IHC learning resources in Italian primary schools.

We carried out the pilot during the second half of the 2019 to 2020 school year in two fifth-grade classes at Matteotti Primary School. We used the Guide for piloting the Informed Health Choices learning resources ^21^ as methodological reference. The pilot study tasks are reported in Table 2, and a Gantt chart is presented in Table 3.

**Table 2.** Pilot study tasks.

*Tasks*	*Participants*	*Activities*
**1. Establishment of the IHC Florence Working Group**		
1.1 Establishment of the coordination group	Researchers	Group responsible for planning, coordinating, and monitoring the steps of the pilot study
1.2 Establishment of the advisory group	Researchers Teachers School principal Family representatives Translators Publisher	Group responsible for reviewing and advising during the pilot study steps
**2. Protocol development**		
2.1 Protocol development	IHC Florence Working Group	Develop the study protocol, request the approval to the Ethics Committee
**3. Preparation of the school intervention**		
3.1 Selection of the school	Coordination group	Select one primary school in Florence
3.2 Selection of the students and teachers	School principal and coordination group	Select two 5 ^th^ year classes of primary school students (10 to 11 years old) and teachers
3.3 Compilation of the informed consent forms	Teachers	Request students’ families and teachers to give their informed consent
3.4 Translation of the IHC learning resources	Researchers	Translate the Informed Health Choices Book and the Exercise Book
3.5 Feedback collected by two teachers and ten children about the Italian translation of the IHC learning resources	Researchers Teachers Students	Send the Italian translation of the IHC learning resources to two teachers and ten fifth grade children, both not involved in the lessons and collect feedback through a questionnaire
3.6 Printing of the Italian translation of IHC learning resources	Publisher (Il Pensiero Scientifico Editore)	Print, without charge, a limited edition of the IHC learning resources translated in Italian language
3.7 Delivery the IHC resources to the school	Coordination group	Send a Children Book and an Exercise book for every student and for every teacher
**4. Intervention in the school**		
4.1 Workshop with the teachers	Researchers Teachers	Introduce the IHC resources and the pilot study; planning lessons to the students and parallel projects (in humanistic, English, art, science areas)
4.2 Lessons to the students	Researchers Students	Teach students to assess treatment claims and make informed health choices with IHC resources
**5. Data collection**		
5.1 Assessment of the IHC resources before the lessons	Teachers Researchers	Critical appraisal of the IHC learning resources by the teachers
5.2 Semi-participatory observations during the IHC lessons to the children	Teachers Researchers	During the lessons evaluate the implementation of the IHC resources and the students’ experience with the resources
5.3 Focused conversations with the students after the lessons	Students Researchers	Guided conversations to explore, through simple questions, the student experience with the IHC resources and lessons
5.4 Assessment of the lessons by the researchers after each lesson	Researchers	Fill in the IHC lesson observation form after each lessons
5.5 Assessment of the lessons by the teachers after each lesson	Teachers	Notes written by the teachers after each lesson
5.6 Assessment of the treatment claims by the student after each lesson	Students Researchers Teachers	Students fill in the Exercise Book and answer oral questions about treatment claims
5.7 Treatment claims assessment by the students at the end of the lessons	Students	Students fill in the IHC Claim Evaluation Tool; oral evaluation of treatment claim
**6. Data analysis**		
6.1 Data analysis	Researchers	Quantitative and qualitative analyses of data
6.2 Formulation of the recommendations	IHC Florence Working Group	Suggest some recommendations on how to implement the IHC resources in the Italian school curriculum
6.3 Dissemination of the results	Pensiero Scientifico Editore, Associazione Alessandro Liberati-Cochrane Affiliate Centre	Publish in a peer-revied journal, disseminate though media and social media

**Table 3.** Gantt chart of piloting activities.

Tasks	Participants	2019												2020												2021					
		1	2	3	4	5	6	7	8	9	10	11	12	1	2	3	4	5	6	7	8	9	10	11	12	1	2	3	4	5	6
**1. Establishment of the IHC Florence Working Group**																															
1.1 Establishment of the coordination group	*Researchers*		X	X	X	X	X	X	X	X	X	X																			
1.2 Establishment of the advisory group	*Researchers Teachers, School principal, Family representatives, Translators, Publisher*				X	X	X	X	X	X	X	X																			
**2. Protocol development**																															
2.1 Protocol development	IHC Florence Working Group		X	X	X																										
**3. Preparation of the school intervention**																															
3.1 Selection of the school	*Coordination group*		X	X																											
3.2 Selection of the students and teachers	*School principal and coordination group*						X	X																							
3.3 Compilation of the informed consent forms	*Teachers*												X	X																	
3.4 Translation of the IHC learning resources	*Researchers*		X	X	X	X	X	X	X	X	X	X																			
3.5 Feedback collected by 2 teachers and 10 children about the Italian translation of the IHC learning resources	*Researchers* *Teachers* *Students*					X	X																								
3.6 Printing of the Italian translation of IHC learning resources	*Publisher (Il Pensiero Scientifico Editore)*											X	X	X																	
3.7 Delivery of the IHC resources to the school	*Coordination group*												X	X																	
**4. Intervention in the school**																															
4.1 Workshop with the teachers	*Researchers* *Teachers*						X																								
4.2 Lessons to the students	*Researchers* *Students*													X	X	X			X												
**5. Data collection**																															
5.1 Assessment of the IHC resources before the lessons	*Teachers* *Researchers*						X	X	X	X	X	X	X																		
5.2 Semi-participatory observations during the IHC lessons to the children	*Teachers* *Researchers*													X	X	X			X												
5.3 Focused conversations with the students after the lessons	*Students* *Researchers*													X	X	X															
5.4 Assessment of the lessons by the researchers after each lesson	*Researchers*													X	X	X			X												
5.5 Assessment of the lessons by the teachers after each lesson	*Teachers*													X	X	X															
5.6 Assessment of the treatment claims by the students after each lesson	*Student* *Researchers* *Teachers*													X	X	X															
5.7 Treatment claims assessment by the students at the end of the lessons	*Students*																		X												
**6. Data analysis**																															
6.1 Data analysis	*Researchers*																					X	X	X	X	X					
6.2 Formulation of the recommendations	*IHC Florence Working Group*																									X	X	X	X	X	X
6.3 Dissemination of the results	*Pensiero Scientifico Editore, Association Alessandro Liberati Cochrane affiliate centre*																													X	X

***School selection***

Matteotti Primary School (part of Istituto Comprensivo Poliziano) is a public school in Florence that is open to external projects and settled in a mixed socio-economic context. This school was selected by convenience sampling. In June 2019, CA and RR presented the project in person to the school principal and then to the collegial school bodies. After being approved by all the bodies, the project was introduced among the external projects for the 2019 to 2020 school year. Ten hours for each participating class were allocated to this project.

***Class selection***

The school principal selected two fifth-grade classes to participate. These two classes were selected by exclusion: classes with either a reduced course load or that were delayed in teaching school curriculum subjects were excluded.

***Participants***

A total of 46 children (aged 10–11 years) from two fifth-grade classes, four of their teachers, and two physicians (CA and RR) participated the piloting. CA and RR led the lessons. The four participating teachers were regularly in charge of the two selected classes. They performed the role of outside observers but also had a supporting role during the lessons (semi-participant observation).

***Ethics approval and informed consent***

We obtained an approval exemption for the study protocol from the Paediatric Ethics Committee of the Meyer Hospital in Florence, as no patients, biological specimen or clinical data were involved in the project. We obtained informed consent (extended data - S1 File) ^42^ for piloting from the schoolteachers and children’s families prior to the onset of the pilot.

***The pilot intervention***

The intervention included the following activities:

• Meeting with the schoolteachers before the beginning of the lessons
• Pre-lesson assessment using Claim Evaluation Tool
• Teaching nine lessons
• Post-lesson assessment using Claim Evaluation Tool

***Meeting with the teachers***

Before the lessons we met with the teachers to introduce them to the IHC learning resources and schedule the lessons. We gave each teacher a copy of the Health Choices Book, Italian translated version. ^25^

### Teaching the lessons

One week before starting the lessons, CA and RR delivered 46 paper copies of the Claim Evaluation Tool (Italian translation) to the teachers. The teachers administered the Claim Evaluation Tool to the children and explained to them that it was a preliminary questionnaire before the project started. From the second half of January 2020 to the first half of June 2020, CA and RR taught nine lessons to each of the two classes. Lessons were scheduled to finish in mid-April, but were interrupted in March after Lesson 7, because of the national lockdown due to the SARS-CoV-2 pandemic. We restarted lessons in May and taught Lessons 8 and 9 remotely through Google Meet.

Before the start of the lessons, CA and RR gave each child a copy of the Health Choices Book (Italian translated version). Every lesson was focused on a chapter of the *Health Choices Book.* We performed a further online meeting with the children during which we conducted individual oral interviews with each child. During each lesson, at least one schoolteacher was present, both to observe and support interactions with the children.

CA and RR taught the lessons according to the *Teachers’ Guide.* During the first 5 minutes of each lesson, we reviewed the previous lesson through a questions and answers session. During this time, we also addressed the most frequent mistakes that we had detected while correcting *Exercise Books* from the previous lesson, by explaining the topic again and using additional examples as needed.

During the next 10 minutes of the lesson, we explained the keywords that are at the beginning of each chapter of the *Health Choices Book.* Then, we introduced the lesson’s topics in two ways: by asking questions to the children and by using real-world examples. For example, before Lesson 5 (“Comparisons of Treatments”), we asked the children how, in their opinion, health researchers could build a good basis for a health claim. After a brief discussion, some children in both classes suggested that a comparison was needed to form a good basis. After this phase, we used the story of James Lind and scurvy ^26^ as vivid example of why we need to make comparisons to determine the effectiveness of treatments. In other lessons, we provided examples not found in the *Health Choices Book*, such as the case of hormone replacement therapy ^27^ to help explain why people included in a treatment comparison should not decide which treatment they got, the ORBITA trial ^28^ to delve into the placebo effect, and sham procedures. Since the story of James Lind attracted children’s attention, we ran other examples from real clinical trials and narrated these examples to the children as stories.

In Lessons 8 and 9 (taught remotely), we used the theme of COVID-19 to explain how study groups that are too small or studies without a proper control group can provide unreliable results. We discussed the case of hydroxychloroquine in COVID-19. ^29^

Following this introduction phase, we read a chapter from the *Health Choices Book* for 15 minutes, where CA was the voice of female characters and RR that of male characters. Then we facilitated class discussion by asking questions and exploring real-life examples.

In the next 15 minutes, we guided the class through group activities related to each chapter. Then the children filled in exercises in the *Exercise Books* and returned them to us for correction before the next lesson.

We used the last 15 minutes of each lesson for questions, comments, and feedback through focused conversations ^30^ with the children. After each lesson, CA and RR noted comments and observations from the schoolteachers and filled in observation forms (extended data - S7 File). ^42^

At the end of the lesson cycle, right before the upcoming school year ended, we gave students access to the Claim Evaluation Tool through the online school platform, with instructions to fill in the questionnaire alone and send it by email to CA. We sent email reminders to students during the following weeks.

### Data collection

***Translation feedback***

The Italian translation finalized draft of the *Health Choices Book* was subjected to two rounds of feedback. In the first round, two teachers not involved in the project (a former humanities teacher in secondary school and a primary school teacher) provided feedback. CA and RR selected the teachers by sampling convenience and based on these teachers’ wide experience in school projects and as teacher trainers. We gave the teachers a copy of the draft and asked them to respond to a 6 items questionnaire (extended data - S2 File) ^42^ in addition to writing a comment about their general impressions of the translated IHC resources (extended data - S3 File). ^42^

In the second round, we collected feedback from 10 children, ages 10 to 11 years, who attended the fifth grade in Matteotti Primary School, and who were not scheduled to participate in the upcoming lessons of the IHC project. One of the fifth-grade classes was randomly selected by the vice-principal and the class teacher selected 10 children based on a progressive gradient of reading and comprehension skills. We sent a letter to the selected children and their parents asking each child to read one chapter of the latest version of the *Health Choices Book* (Italian translation) and to answer three questions about the text (extended data - S4 File). ^42^

***Pilot data collection***

To analyse the five pilot objectives, we used seven different methods of data collection (for an overview of what data informed which objective, see Table 4):

1. *Teachers’ semi-participatory observations after each lesson.* At least one schoolteacher attended each lesson. After each lesson CA or RR had a brief meeting with the teachers and conducted semi-structured interviews with them, based on three open questions: 1) Do you have any observation about the lesson you have attended? 2) How do you think the children dealt with the Key Concepts? 3) Do you have any suggestion for improvement? CA or RR made written notes from the teachers’ answers after the meeting.
2. *Feedback at the end of each lesson by CA and RR.* After leading each lesson, CA and RR filled in the observation form, which is provided in the “Guide for Piloting the Informed Health Choices Learning Resources” ^21^ (extended data - S7 File and S8 File). ^42^
3. *Children’s focused conversations after each lesson.* CA or RR moderated these focused conversations that included four categories of questions, referred as ORID set of questions ^31^: Objective (to collect information about the context): “What was this lesson about?”; Reflective (to identify feelings associated with information): “Was there anything less easy to understand? What did you like most? What did you dislike?”; Interpretive (what it means to you): “Have you got any insights or comments about the lesson concepts?”; Decisional (what are the next steps): “What would you improve or change in the lesson or in the textbook?” We spoke to the children as a class. CA or RR (whoever was not moderating the conversation) collected data through written notes during the session (see extended data - S10 File). ^42^
4. *Children’s answers to exercises in the Exercise Book for Lessons 1 to 7* (not feasible for Lessons 8 and 9 which were taught remotely). After each lesson, each student filled in the corresponding exercises in their Exercise Book and handed them in to CA and RR. We corrected and returned them to the children at the beginning of the next lesson. Exercises included true/false statements, requests to define terms (e.g. “What is a claim?”), and detection activities (e.g., identify the basis of a provided health claim).
5. *Individual children’s interviews after the end of the lesson cycle.* After the lessons’ cycle, CA or RR interviewed children individually, asking one question to each child. These questions were designed to assess knowledge of the Key Concepts and ability to apply this knowledge to real or hypothetical situations. Each child answered one different question that we chose randomly (extended data - S13 File), ^42^ and we made written notes of their answers.
6. *Teachers’ evaluation of the IHC learning resources at the end of the lessons cycle.* After the end of the lessons’ cycle, we explored the teachers’ experience of the IHC learning resources. We emailed them an evaluation questionnaire (see extended data - S11 File and S12 File). ^42^ Each teacher filled in the questionnaire and emailed it back to us.
7. *Children’s answers to the Claim Evaluation Tool questionnaire, both before and after the lessons’ cycle.* The objective was to address the ability of the students to assess health claims about treatments and make informed health choices. After the end of the lessons, the Claim Evaluation Tool was uploaded to the online school platform. The questionnaire included 24 questions (15 multiple-choice questions and 9 true/false statements). The students were asked to fill in the questionnaire alone and send it via email. We collected and anonymized the data.

**Table 4.** Pilot study objectives and data collection.

*Study objectives*	*Data collection*						
	Evaluation of the results of Claim Evaluation Tool	Semi-participative observation by the teachers during the lessons	Feedback about the lessons using observation forms (CA and RR)	Focused conversations with children at the end of each lesson	Evaluation of the exercises completed by the children during each lesson	Final individual interview with each child	Evaluation of the IHC resources by the teachers after the 10-lessons cycle
Feasibility of introducing IHC curriculum in Italian school	**X**	**X**	**X**	**X**	**X**	**X**	**X**
Students’ ability to assess health claims	**X**	**X**	**X**	**X**	**X**	**X**	
Students’ experience with the IHC resources (understandability, desirability, suitability, and usefulness)	**X**		**X**	**X**	**X**	**X**	
Teacher’s experience with the IHC resources (understandability, desirability, suitability, and usefulness)		**X**	**X**				**X**
Facilitators and barriers	**X**	**X**	**X**	**X**	**X**	**X**	**X**

### Data analysis

***Translation feedback: qualitative analysis***

We analyzed the data using a thematic analysis approach. ^32^ After familiarizing ourselves with the translation feedback, CA and RR categorized the feedback through six thematic labels already used for the evaluation of teachers’ feedback about the IHC resources ^20^: errors in the text; expressions or concepts not understood; expressions or concepts that can improve their understanding; additional text suggestions to improve understanding; general comments to improve resources; and errors in the edition. We coded all the answers from the teachers’ 6 items questionnaire (extended data - S2 File) ^42^ according to these themes, except for the final comment, attributed these data to the six thematic labels and made a summary report. Then, we analyzed the teachers’ final comments through a deductive thematic analysis based on categories teams have previously employed when exploring user experience of the IHC learning resources ^19^^–^^21^: understandability, desirability, suitability, and usefulness.

We independently assigned each data to a user experience category and resolved disagreements through a reassessment of the attributions until a final agreement was reached. Then, we generated a final narrative summary (extended data - S3 File). ^42^

We coded the children’s answers to the interview questions using three thematic labels based on categories teams have previously employed exploring children’s user experience of IHC learning resources ^20^: what children liked about the text, what children did not like about the text, difficult words that were highlighted and generated a narrative summary of this feedback (extended data - S5 File). ^42^

***The pilot intervention: quantitative analysis***

For the children’s answers in the *Exercise Books*, we assessed the mean proportion of correct answers for each chapter *,* the mean proportion of correct answers in chapters 2, 3 and 4 (content focused on bad bases of health claims) and in chapters 5, 6 and 7 (content focused on comparisons of treatments). For each question in the *Exercise Book*, we also assessed the proportion of answers with less than 70% correct rate.

To analyse the Claim Evaluation Tool answers, both pre- and post-intervention, we assessed the mean score and standard deviation (SD) of the proportion of correct answers at the individual level; the proportion of the students with a passing score (≥13 right answers out of 24); and the proportion of the students with a mastery score (≥20 right answers out of 24). These cut-off scores for passing (having at least a borderline ability to apply the concepts) and mastery (having mastered the concepts) were determined in a previous study, using judgments made by researchers and teachers in a combination of established methods to reach consensus. ^33^

***The pilot intervention: qualitative analysis***

The following qualitative data were collected: (1) Teachers’ semi-participatory observations after each lesson; (2) Feedback at the end of each lesson by CA and RR; (3) Children’s focused conversations with CA and RR after each lesson; (4) Individual children’s interviews after the end of the lessons; (5) Teachers’ evaluation of the IHC learning resources at the end of the nine lessons cycle.

We conducted a deductive thematic analysis ^32^ of the collected data based on the categories previously employed in IHC project pilots ^19^^–^^21^: user experience (understandability, desirability, suitability, and usefulness), seriousness of these experiences for the user, teaching method, barriers and facilitators, proposals, and comments. CA and RR organized transcribed interviews of children and teachers in two files. Then we independently coded the data according to categories. For each category, we discussed attributions, and dealt with disagreements through extended discussion. Data that we could not place into prespecified categories were coded as “Comments”. Finally, we created a narrative summary of the data for each category and explored the range and nature of the phenomenon, as well as some possible explanations for the results.

Regarding the final individual children interview, we evaluated children’s answers based on three categories related to learning the IHC Key Concepts: assessing the reliability of health claims about treatments’ effects; assessing if a comparison between treatments is fair; knowing how to make an informed health choice. We defined the first and second objective as ‘Knowledge’ and the third objective as ‘Orientation’. ^34^ Moreover, we evaluated students’ learning of each objective based on four descriptive grades of evaluation in accordance with those indicated by the Italian Ministry of Education for the learning evaluation in primary schools: advanced, intermediate, basic, in the process of first acquisition (extended data - S9 File). ^42^

## Results

### Adaptation of the Italian translation

No major difficulties or issues were encountered with the Italian translation of the learning resources. However, we made several choices that are explained below:

• The title “The *Health Choices Book*” has been translated into the Italian equivalent of “ *Health Decisions Book”.* Choice and decision are not true synonyms in Italian: from an etymological perspective, both terms refer to the concept of “selection” ( *ex-legere* and *de-caedere* in Latin), but from a semantic perspective, their meaning is different. “Choice” refers to the power or capability to choose, whereas “decision” refers to determination that is achieved after examining the available information. As the objective of the IHC project is to teach how to make health decisions that are informed by an adequate evaluation of the available evidence and how this evidence matches with an individual’s values and preferences, we opted to use the term “decision” instead of “choice —in line with the Spanish and French translations.
• The first names of professors “Connie Compare” and “Francis Fair” were omitted, and only their translated surnames were used, such as in the Spanish translation. Moreover, in Italian, there is no alliteration between names and surnames, which is present in English. The names have, therefore, been translated as “Professoressa Confronto” (English translation: Professor Compare) and “Professor Giusto” (English translation: Professor Fair).
• In the original text, some keywords are provided in Kiswahili, the state language of Uganda, to aid understanding. We substituted with these with English terms in the Italian translation. As English language is a curricular subject in Italian primary schools, this provided children with an extra opportunity for language learning.
• Feedback from teachers and children about the draft translation of the *Health Choices Book* are presented in S2 File, S3 File and in S5 File (see extended data). ^42^ Teachers found no errors or comprehensibility issues over the text. They both found the text well written, the story compelling and age appropriate for the children. They made suggestions about how to convey some contents of the text to the children. One teacher stressed the need to explain to the primary school children a broader meaning of “personal experience”: in the *Health Choices Book* personal experience is presented as an incorrect basis for a claim about a health treatment’s effect. However, in other areas, personal experience can be a vehicle for identity and growth because it informs on the personal impact with a life event. Another teacher pointed out that, while learning the Key Concepts, children should be considered as individuals within a specific context (both the class and the family context). “The family, in particular, should be involved through various initiatives”.

All children rated the text as interesting and fun and did not raise any major issue. Challenging terms that children understood less well are “mislead”, “herbalist”, “malaria”.

CA and RR made minor changes to the draft after according to teachers’ and children’s feedback, and a final version was completed in the autumn of 2019.

In December 2019, an Italian publisher—Il Pensiero Scientifico Editore—printed 100 copies of a limited edition of the *Health Choices Book* ^25^ and 60 copies of the *Exercise Book* for free.

***Quantitative analysis***

**Figure 4.**
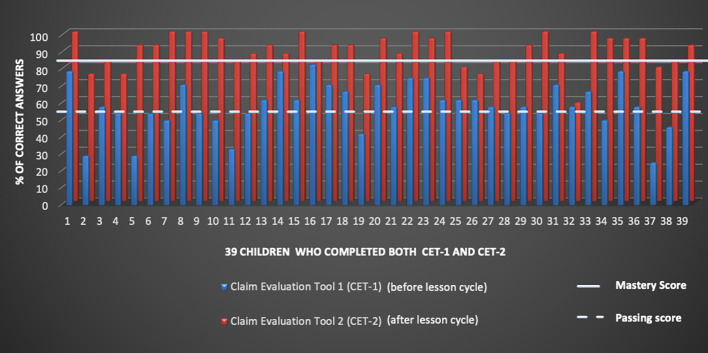
Individual percentages of correct answers for 39 children who took Claim Evaluation Test 1 (before lesson cycle) and Claim Evaluation Test 2 (after lesson cycle).

The main results of quantitative analysis are summarized in Table 5, Table 6, and Figure 4.

**Table 5.** Quantitative analysis: Exercise Book and Claim Evaluation Tool.

**Exercise Book**	**Chapters 2,3 and 4** «Bad Bases of Health Claims»	**Chapters 5,6 and 7** «Treatment Comparisons»
Mean proportion of correct answers (SD)	**88.3%** (4.6)	**83.2%** (7.8)
**Claim Evaluation Tool**	**Claim Evaluation Tool-1** (n=45/46 children)	**Claim Evaluation Tool-2** (n=40/46 children)
Mean proportion of correct answers (SD)	**56.3%** (16.6)	**89.2%** (9.4)
Proportion of students who achieved a passing score (≥13 correct answers out of 24)	**71.1%** (32 of 45 children)	**100%** (40 of 40 children)
Proportion of students who achieved a mastery score (≥20 correct answers out of 24)	**2.2%** (1 of 45 children)	**82.5%** (33 of 40 children)

**Table 6.** Exercise book: % of correct answers from responding children (for each lesson).

Lesson	n. (%) respondents	Mean % correct answers (standard deviation)
**One**	43 (93.5%)	86.0% (10.7)
**Two**	43 (93.5%)	86.7% (6.7)
**Three**	39 (84.8%)	94.5% (3.7)
**Four**	42 (91.3%)	83.6% (9.8)
**Five**	42 (91.3%)	87.8% (9.6)
**Six**	40 (86.9%)	72.2% (20.4)
**Seven**	45 (97.8%)	89.7% (8.5)

***Exercise book answers***

A total of 46 students attended the lessons (two classes with 24 and 22 children, respectively) and participated to either CET-1 or CET-2. Twenty-eight (60.9%) were female. All children were 10 to 11 years old. 91.3% of the students completed the exercises included in the *Exercise Book*, except for Lessons 8 and 9 that took place remotely.

For Lessons 2 to 4, which focused on the need to assess inadequate bases of health claims, the mean proportion of correct answers was 88.3% (DS 4.6). For Lessons 5 to 7, which focused on comparisons of treatments, the mean proportion of correct answers was 83.2% (DS 7.8) ( Table 5 and Table 6).

The lesson showing the least proportion of correct answers (72.2%) was Lesson 6, which was about fair treatment comparisons.

We also analysed the proportion of correct answers for each question, with the following questions resulting in less than 70% of the children with correct answers:

- 35.0%: “Should the people in the comparison choose who gets the vaccine? Why?” (Lesson 6, page 34, question 1)
- 47.5%: “In a fair comparison, the treatment is the only important difference between groups” (Lesson 6, page 32, true/false statement 3)
- 58.1%: “This book tells you what treatments to use” (Lesson 1, page 7, true/false statement 2 of exercise 2)
- 62.5%: “Should the professor choose who gets the vaccine? Why?” (Lesson 6, page 33, exercise 2, question 2)
- 69%: “The basis for the claim is more important than who is making the claim” (Lesson 4, page 20, exercise 1, true/false statement 4)

See also extended data - S14 File. ^42^

***Claim evaluation tool***

Out of 46 children, 45 (97.8%) completed CET-1 and 40 (87.0%) completed CET-2. The mean proportion of right answers for all children was 56.3% (SD 16,6) for CET-1 and 89.2% (SD 9.4) for CET-2.

The proportion of students who achieved a passing score (≥13 correct answers out of 24) was 71.1% (32/45) for CET-1 and 100% (40/40) for CET-2. The proportion of students who achieved a mastery score (≥20 correct answers out of 24) was 2.2% (1/45) for CET-1 and 82.5% (33/40) for CET-2 ( Table 5).

Thirty-nine students completed both CET-1 and CET-2. Among these students, the mean difference in the proportion of correct answers between CET-1 and CET-2 was 30.1% (95% CI 25,5%-34,8%; p< 0.00001). The mean difference in the proportion of those achieving a passing score was 23.1% (95% CI 9.2%-36.9%), while the mean difference in the proportion of those achieving a mastery score was 79.5% (95% CI 66.2%-92.7%). Thirty-seven students out of 39 (94.9%) improved their score from CET-1 to CET-2, while 2 students out of 39 reached the same score in the two tests. No student worsened between CET-1 and CET-2 ( Figure 4).

See also extended data - S15 File. ^42^

### Qualitative analysis

The results of the qualitative analysis are summarized with more detail in S6 File (see extended data). ^42^

***Seriousness for the users***

Neither the students nor the schoolteachers reported any major or minor issues during the lessons. The teachers pointed out that the *Health Choices Book* is written with an adequate language for these students’ age and that drawings and dialogues among characters further help comprehension and positively attract students’ attention. Moreover, the teachers underlined positively how the book’s topics are dealt with according to a gradient of difficulty.

The students unanimously observed that the comic makes it easier and enjoyable to learn concepts and that most examples made were consistent with their everyday lives.

The schoolteachers underlined the relevance of the first chapter, which lays the foundation for many concepts that are addressed over the subsequent chapters. One teacher suggested splitting the first chapter into two lessons to facilitate learning.

***Understandability and desirability***

Both the students and the schoolteachers found the understandability of the IHC learning resources to be good. They highlighted how the subdivision of episodes helped the children isolate each topic and understand it better. Moreover, the teachers observed that the questions in the *Exercise Book* offered the children a moment of self-assessment and that group activities promoted a consolidation of what had been learnt. RR and CA noted that the main difficulties were about learning language children are not used to. For example, the distinction between claim, basis, treatment, and effect can be difficult at first, but the structure of the book with repeated examples and activities helped children to increasingly master these concepts.

Some students observed that the keywords at the beginning of each chapter were not always easy to understand at first (such as “chance” or “claim”), but they were subsequently fully clarified by examples provided in the *Health Choices Book.*

Regarding desirability, the students and schoolteachers valued it as excellent. They did appreciate the comic, characters, and dialogues. Most of the students appreciated learning about the bad bases of treatment claims. From the beginning, the students were very interested in the Key Concepts and enthusiastic about the lessons. This positively affected their motivation to learn.

***Suitability***

We also found suitability to be high. Concepts explained in the *Health Choices Book* elicited examples from the students’ daily lives. For example, the “new is better” concept inspired a discussion among the children about the differences between branded and non-branded products. Moreover, they discussed the widespread claim that carrots are good for sight while learning the concept that “widely used treatments or those that have been used for decades are not necessarily beneficial or safe”.

None of the children raised issues about the location of the *Health Choices Book* (the book story takes place in Africa, and the main characters are two students at a local school). On the contrary, as a schoolteacher pointed out, the different setting and unusual sound of some African names constituted an added value for the children’s interest and curiosity. Furthermore, the teachers also observed that although the book is set in a context that differs in some ways from the children’s own, the situations, dialogues, and concepts in the book are universal and independent from the context.

***Usefulness***

**Figure 5.**
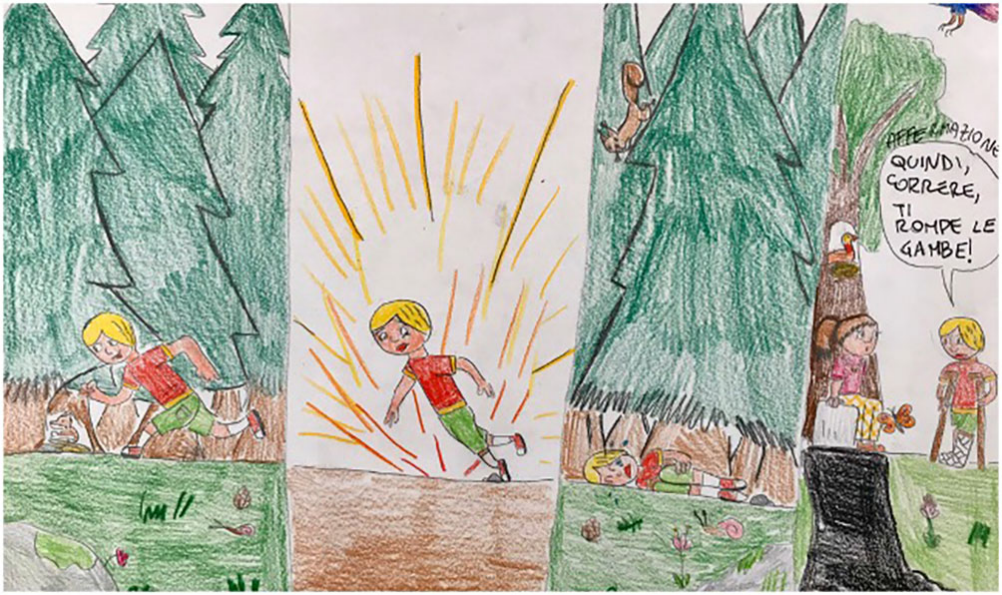
Drawing from a child explaining the Key Concept of “Personal Experience”.

Regarding the usefulness of the Key Concepts, the schoolteachers used these concepts to integrate the core curriculum of the students with new competencies, such as a project about the critical appraisal of advertisements. Moreover, the children were enthusiastic about transposing the learned concepts to drawings and to examples from their daily lives (e.g. the concept of personal experience as a bad basis for a treatment claim) ( Figure 5).

***Facilitators and barriers***

*Value of the IHC learning resources*

Both the students and the schoolteachers highlighted the value of the IHC learning resources. The students appreciated the comic and realistic examples and found the activities proposed in the *Exercise Book* to be very entertaining.

The schoolteachers underlined the value of the IHC learning resources in terms of the ability of these resources to teach critical thinking. The kind of comic, way of teaching the lessons, and group activities were considered facilitators of the learning of the Key Concepts.

Finally, the schoolteachers attending the lessons underlined how class participation was widespread and did not involve only the most enterprising students.

*Compatibility with the Italian school curriculum and with the teaching style of teachers*

The development of critical thinking is deemed a cross-cutting competence between the various topics of the individual school curriculum. Importantly, we discovered that teachers used the Key Concepts in parallel projects in other subjects. For example, building on the concepts about the bad bases for health claims, one teacher started a project about taking a critical approach to advertisements. The teacher selected some advertisements making claims about food and about oral supplements. The students, organized in small groups, had to analyze both the design and the content of each advertisement, and identify the sender and receiver of the message, the claim of the advertisement and the basis of the claim. Then a representative from each group reported the responses to the class and the children discussed the reliability of the advertising message.

Another teacher upgraded the science topic "human body" through the exploration of treatments’ effects about illnesses of organs or systems. This teacher asked CA and RR to correlate some of the “human body” topics to some Key Concepts of the IHC lessons. For example, we used the topic of the musculoskeletal system to explain to children the concepts of placebo effect and shame procedures regarding meniscus degeneration treatments and described how this was investigated in clinical trials.

Finally, another teacher asked the children to transfer the learned Key Concepts into drawings. The transposition of the concepts into images helped the children focus on what they had learned ( Figure 5), and it also helped us discover which students did not completely understand a given concept.

Moreover, teachers pointed to additional potential areas of projects parallel to the IHC curriculum: e.g., in mathematics, civic education, and technology (extended data - S6 File). ^42^

The teachers observed that the resources were compatible with their usual teaching method. They valued interactivity of the lessons and the employment of practical examples.

*Incentives and disincentives*

The teachers underlined that support from the school principal and the possibility of connecting the IHC learning resources to the school curriculum were incentives for the project implementation. They also cited the school’s openness to external projects and the possibility of using the interactive whiteboard to visualize the learning resources as incentives for project realization.

The only disincentive that was highlighted by both the students and the schoolteachers was remote learning in the later lessons. The teachers pointed out that remote learning hindered interaction with and within students, with a negative impact on lessons participation: teachers observed that lessons’ engagement was mostly reduced in children with lower school performance.

Children’s attendance was also lower for the online lessons. CA and RR observed that technical issues during online lessons (e.g., internet connection, microphone malfunctioning) often hindered interaction with the students and within the students.

***Transfer to real life experience***

All the teachers observed that learning about the Key Concepts raised their awareness about how unreliable health claims have the power to mislead health choices and how it is crucial to become able to recognize the correct basis of a health claim. Teachers were impressed by the concept that expert opinions are not a good basis for a health claim if these opinions are not grounded on fair comparisons.

They also pointed out that, after attending the IHC lessons, they felt more confident in asking their physicians questions about the potential adverse effects of treatments and about the option of not using a treatment at all during an illness.

During the lessons, the students mentioned and reconsidered many claims that they had been exposed to (e.g., through advertisements or through friends’ and relatives’ claims), even outside health science. For example, one child described making a comparison between a set of branded and expensive crayons with a cheaper and not-branded set: she coloured two drawings with these two crayons sets and didn’t observe any major difference in the final effect.

***Final oral interview with children***

A qualitative evaluation of the final oral interview with children is reported in S9 File (see extended data). ^42^

We found that 30 out of 44 students (68.2%) had an advanced knowledge of the Key Concepts and 30 out of 44 (68.2%) had an advanced orientation. These results confirmed that most students acquired a good knowledge and a good orientation about the Key Concepts presented in these resources and skills to begin think critically about health claims.

## Discussion

### Major findings

Findings from this first study about the contextualization of the IHC learning resources in Italian primary school indicate that these resources are compatible with the Italian primary school context and with the Italian primary school curriculum. Results of the quantitative and the qualitative analyses consistently showed positive experiences with the IHC learning resources in both children and teachers.

These findings are consistent with those reported in other contextualization studies of the IHC learning resources in different countries. ^35^ Like other IHC pilot experiences in Europe, the unfamiliar African village setting of the story in the *Health Choices Book* was not an obstacle to the children’s interest. Instead, it appeared to add value, as the story set in a different place from children’s usual life context seemed to stimulate children’s curiosity. Furthermore, although the *Health Choices Book* sometimes refers to common practices that are unusual for the Italian cultural context (e.g., putting cow dung on burns), some of these are comparable to Italian local practices (e.g., putting oil of olive on burns), and therefore did not constitute a barrier for text comprehension.

A novel result of this study was the suitability of the Key Concepts to be applied to other knowledge areas in primary school: for example, in parallel with the IHC lessons, the schoolteachers started projects about taking a critical approach to advertisements and creating figurative representations of the Key Concepts. Although, to our knowledge, this finding has not been reported yet in other IHC pilot experiences in primary schools, the IHC Key Concepts have been transposed to fields outside health science, such as education and environmental policies, ^12^ and can therefore provide a common conceptual map for thinking critically about effect claims across subjects.

### Strengths and limitations

This study has important limitations. First, it involved a small number of children belonging to a single primary school, which weakens the generalizability of the results. Second, unlike the Ugandan trial, two physicians (CA and RR) taught the lessons to the children, rather than the schoolteachers. CA and RR also gathered the data and were part of the research group that analysed them. It is uncertain to what extent this involvement could have influenced the lesson’s delivery, as well as the data collection and analysis. However, the Ugandan randomized trial found that the use of these learning resources improved children’s ability to assess health claims, even when taught by teachers with no medical background. ^17^ Therefore, we can hypothesize that having teachers with medical background lead the lessons did not constitute the key element for this piloting’s results.

Third, the very positive results from the Claim Evaluation Tool may not be reliable. In this study, not only all the students involved in the IHC lessons obtained a passing score on the Claim Evaluation Tool final questionnaire, but 80% of them obtained a mastery score, i.e., they mastered the Key Concepts. However, there was no control group for the assessment of CET results, and the CET was administered to the children both before and after the lessons. The before-after test administration could have given an advantage to the children and be ultimately responsible for an overestimation of the final scores.

However, the primary objective of this piloting was not to demonstrate the ability of the IHC learning resources to improve critical thinking about health claims in primary school children —an objective already met in a large randomized controlled trial— but to evaluate to what extent these resources could be integrated into the Italian school curriculum. The dramatic difference in Key Concepts learning we found before and after the lessons is an indirect indication that these contextualized resources are understandable and suitable for teaching Italian primary school children.

An important strength is the consistency of the feedback received from different sources and gathered through heterogeneous methods. The results of both the qualitative and quantitative analyses converge, underscoring the feasibility of implementing contextualized IHC learning resources in Italian primary school.

### Content that was more difficult

The mean percentage of correct answers from the *Exercise Book* was more than 80%. However, some concepts were found to be more difficult to understand for the children. For example, a few questions about chapters 4 and 6 had the highest rate of errors. In these chapters, the concepts of blinding participants and blinding personnel within a fair comparison was particularly difficult to understand for children. Whether confirmed in future pilot studies, these findings could be useful for teachers to be aware of.

### Challenges with remote learning

All teachers reported remote learning as the only barrier detected for the implementation of the IHC learning resources. In March 2020, Italy was the first country in Europe to have a national lockdown and the resulting closure of all grade schools led to a shift to remote learning. Although remote learning has been an opportunity to carry on the school programs and to maintain contact between teachers, children and families, research has pointed out also its downsides. In a survey, which was carried out between October and November 2020 in eight countries, more than 2,500 teachers were asked about the effectiveness of online schooling. Overall teachers gave remote learning an average effectiveness of five out of ten. Moreover, they observed some students had a learning delay of around three months and that those from poorer backgrounds were falling further behind. These observations are consistent with our experience: during our piloting, both we and the teachers observed that the interaction between students and teachers was reduced during the remote lessons. Teachers also observed that this phenomenon occurred to a greater extent in students with lower school performances. Moreover, during the online lessons we were unable to carry out activities at the end of the lesson precisely because the online mode would have greatly limited the interaction necessary for these activities. Although we have replaced the activities with oral questions, we have lost a tool that allowed the children to consolidate the Key Concepts they learned while having fun. Consistently, some studies indicate that active-learning results in better learning outcomes than passive learning or instructor-centered approach, both in-person and online. ^36^^–^^39^

## Conclusions

Health literacy is a *conditio sine qua non* for making good health choices. The SARS-CoV-2 pandemic has further underlined the desperate need for widespread health literacy in the whole population. ^40^ Education and health have common interests in developing students’ ability to think critically about the information they encounter. Acquiring a critical attitude toward health claims starting in primary school can lay the foundations of thinking carefully about health choices later in life, with the potential to impact health outcomes across a wide population group.

In Italy, objectives of the scientific curriculum for primary school entail “detecting phenomena, asking questions, constructing hypotheses; observe, experiment and collect data; formulate conclusive hypotheses and verify them” in order to “raise the logical and critical thinking”. The IHC learning resources satisfy all these objectives and may therefore represent an engaging foothold towards science since they concern health, that is a highly relevant and universal topic. Children can learn the bases of critical thinking about health treatments in primary school and refine these skills as they grow up. That way, as adults, they will be able to make better health decisions for themselves and to successfully participate to an informed public debate.

This pilot study represents a first step for broader contextualization activities aimed at consolidating our results and at fostering the inclusion of IHC concepts in the scientific curriculum of Italian primary schools.

## Data availability

### Underlying data

All data underlying the results are available as part of the article and its supporting information figures and tables.

### Extended data

Zenodo: Feasibility of contextualizing the Informed Health Choices learning resources in Italy: A pilot study in a primary school in Florence. Supporting information, DOI: https://doi.org/10.5281/zenodo.6581224. ^42^

This project contains the following extended data:

- **S1 File. Informed Consent for children’s parents and teachers**
- **S2 File. Teacher’s feedback about the Italian translation of the IHC resources**
- **S3 File. Qualitative analysis of teachers’ final comments about the Italian translation of the Health Choices Book**
- **S4 File. Feedback collection from children about the translation of the Health Choices Book**
- **S5 File. Children’s feedback about the Italian translation of the Health Choices Book**
- **S6 File. Thematic analysis of qualitative data (extended version)**
- **S7 File. Lesson Observation Form IHC**
- **S8 File. Lesson Observation Form IHC Results**
- **S9 File. Results of the final individual interview with the children**
- **S10 File. Focused Conversation with the students**
- **S11 File. Evaluation Questionnaire for Schoolteachers after the end of the 10-lessons’ cycle**
- **S12 File. Teachers’ answers to the evaluation questionnaire of the IHC learning resources at the end of the lessons’ cycle**
- **S13 File. Final individual interview with each child**
- **S14 File. Exercise Book results**
- **S15 File. Claim Evaluation Tool results**

Data are available under the terms of the Creative Commons Attribution 4.0 International license (CC-BY 4.0).

### Reporting guidelines

Zenodo: COREQ (COnsolidated criteria for REporting Qualitative research) Checklist. Feasibility of contextualizing the Informed Health Choices learning resources in Italy: A pilot study in a primary school in Florence, DOI: https://doi.org/10.5281/zenodo.6581224. ^43^

Data are available under the terms of the Creative Commons Attribution 4.0 International license (CC-BY 4.0).

## Author contributions

CA made the draft of the article. RR and SR reviewed the draft. All authors have reviewed the draft. CA and RR codified the categories of the qualitative thematic analysis, and all authors evaluated the attributions to each label. CA, RR and GF performed the quantitative analysis. CA, RR and SR reviewed the final quantitative analysis. CA and SR reviewed the final qualitative analysis. CA and SR revised the final version of the tables and figures.
