# Supplementary material for: Feasibility of contextualizing the Informed Health Choices learning resources in Italy: A pilot study in a primary school in Florence
Source: F1000Res. 2022 Oct 12;11:1167. [Version 1] doi: 10.12688/f1000research.123728.1 (PMC9617069; doi:10.12688/f1000research.123728.1)
Supplement: Peer review history

# Peer review: Reviewer response for version 1

**DOI:** 10.5256/f1000research.135861.r153254

**Published:** 2022-10-28

**Authors:** Biesty Linda

**Affiliations:** 1 School of Nursing and Midwifery, National University of Ireland Galway, Galway, Ireland

This paper was an absolute pleasure to read. The background section and the links to other activity within the IHC Group programme of research are clearly identified. The rational for methods used in this study are offered, the different methods are reported clearly and in a manner that contributes to the rigour of the work. Findings are presented clearly and supplemented with links to allow the read a more in-depth exploration of the audit trail and data that contribute to the findings. Interesting conclusions drawn that are grounded in the strengths and acknowledged limitations of this work, this will be useful across the community for researchers, teachers and the public interested in this space.

Is the work clearly and accurately presented and does it cite the current literature?

Yes

If applicable, is the statistical analysis and its interpretation appropriate?

I cannot comment. A qualified statistician is required.

Are all the source data underlying the results available to ensure full reproducibility?

Yes

Is the study design appropriate and is the work technically sound?

Yes

Are the conclusions drawn adequately supported by the results?

Yes

Are sufficient details of methods and analysis provided to allow replication by others?

Yes

Reviewer Expertise:

Evidence synthesis, qualitative research, midwifery and maternity care, public engagement in research

I confirm that I have read this submission and believe that I have an appropriate level of expertise to confirm that it is of an acceptable scientific standard.

# Peer review: Reviewer response for version 1

**DOI:** 10.5256/f1000research.135861.r153255

**Published:** 2022-10-28

**Authors:** Emanuela Guarcello

**Affiliations:** 1 University of Turin, Turin, Italy

**Summary**

 The article addresses the topic of the promotion of critical thinking about treatments claims and health choices in primary school. Within the frame of this topic, the authors focused on the hypothesis that the improvement of a child's ability to think critically about health choices, starting with primary school, could positively affect their capacity to make good health choices when they will be adults, navigating the overabundance of unreliable or unbalanced information and sharing in a founded way, their health decisions with Physicians.

 The authors found support for this hypothesis in a previous study realized starting from 2012 within the Informed Health Choices (IHC) project, through a randomized controlled trial of 120 schools in Uganda. On the basis of the results obtained in this research, the authors worked to a contextualization of the IHC resources for using them in other languages and settings. In particular, the article describes the contextualization for using them in Italian primary school, with specific regard to assessing the feasibility of introducing the IHC curriculum in Italian primary schools, to evaluate the ability of students in the pilot to assess health claims and make informed health choices, to explore students’ and teachers’ experiences of the learning resources, and to identify barriers and facilitators to the implementation of the IHC curriculum.

**General comments**

 The manuscript is well written, comprehensive and clearly presented, and interesting both for the health and teaching fields. The logic of the submission is cogent, and the presented elements fit well together. In particular, the objectives are clearly and specifically articulated, the theoretical frame is clear, and the literature is indicated and appropriate. The research methods employed, and the data analysis techniques used are appropriate and clear. The sources are clearly defined and well-articulated. The results and the findings are clear, and the final data support the conclusion and the implications that the author states. The findings contribute to advancing our knowledge and understanding with particular regard to the strategies of promotion of critical thinking in primary school, with a special focus on health choices.

**Specific comments**

 Among the main advantages of the presented research, it is possible to identify:

- The well-founded background on which the presented article is placed (randomized controlled trial of 120 schools in Uganda).
- The structure and contents of the resources for teaching, elaborated and tested by the research group.
- The well-articulated methods of data collection and analysis.
- The general research vision, aimed at structuring a systematic curriculum for primary schools in Italy on the promotion of critical thinking (with respect to the topic of Informed Health Choices). This is a point of innovation in the scientific panorama and in current scholastic practices.

Is the work clearly and accurately presented and does it cite the current literature?

Yes

If applicable, is the statistical analysis and its interpretation appropriate?

Yes

Are all the source data underlying the results available to ensure full reproducibility?

Yes

Is the study design appropriate and is the work technically sound?

Yes

Are the conclusions drawn adequately supported by the results?

Yes

Are sufficient details of methods and analysis provided to allow replication by others?

Yes

Reviewer Expertise:

General Pedagogy

I confirm that I have read this submission and believe that I have an appropriate level of expertise to confirm that it is of an acceptable scientific standard.
